# Nuclear Protein in Testis (NUT) Carcinoma: Diagnostic Challenges in a Patient Requiring Rescue Venovenous Extracorporeal Membrane Oxygenation

**DOI:** 10.7759/cureus.113941

**Published:** 2026-08-04

**Authors:** Irvin C Lien, Kshama Bhyravabhotla, Mahmoud M Elsayad, Pushan Jani, Kha Dinh

**Affiliations:** 1 Pulmonary and Critical Care, University of Texas Health Science Center at Houston McGovern Medical School, Houston, USA; 2 Pathology and Laboratory Medicine, University of Texas at Houston, Houston, USA; 3 Internal Medicine, University of Texas Health Science Center at Houston McGovern Medical School, Houston, USA

**Keywords:** fish, fluorescence in situ hybridization (fish), immunohistochemistry in lung cancer, lung cancer, nutm1 gene, thoracic malignancies in young adult

## Abstract

Nuclear protein in testis (NUT) carcinoma is a rare, highly aggressive malignancy defined by *NUTM1* rearrangement, most commonly involving *BRD4*. Nonspecific presentation and poorly differentiated histology lead to frequent misdiagnosis. Patients often present with large mediastinal masses, making swift diagnosis both important and challenging. A 29-year-old woman presented with two months of progressive dyspnea, cough, and fatigue accompanied by new-onset hemoptysis. CT demonstrated a large right hilar and mediastinal mass compressing the trachea, pulmonary artery, and superior vena cava (SVC). Rigid bronchoscopy was planned for biopsy and possible therapeutic debulking to control hemoptysis; the extracorporeal membrane oxygenation (ECMO) team was on standby, but the patient was not pre-cannulated. During induction of anesthesia, she developed profound refractory hypoxemia that persisted despite jet ventilation and airway optimization, attributed to loss of spontaneous respiratory effort and muscle tone with worsening dynamic central airway compression. Emergent femoral-femoral venovenous extracorporeal membrane oxygenation (VV-ECMO) was initiated, allowing the procedure to proceed safely. Bronchoscopy demonstrated severe right mainstem bronchial stenosis and complete obstruction of the right upper lobe by a friable endobronchial mass. Biopsies were nondiagnostic because of extensive necrosis and crush artifact, but the findings defined the extent of endobronchial disease and guided subsequent diagnostic planning. She was decannulated after two days of support, following clinical improvement sufficient to tolerate sitting upright. Supraclavicular lymph node sampling then established NUT carcinoma, confirmed by NUT immunohistochemistry (clone C52B1). Chemotherapy could not be initiated because of her critical clinical state, and she died two weeks after diagnosis. NUT carcinoma presents with nonspecific respiratory symptoms and is usually diagnosed at an advanced stage. Extensive necrosis reduces endobronchial biopsy yield, and alternative tissue sites should be pursued when initial sampling is nondiagnostic. Large mediastinal masses confer high risk of airway collapse and cardiovascular compromise during sedation; this case suggests that pre-emptive cannulation, rather than standby availability, warrants consideration when tracheal, pulmonary artery, and SVC compression coexist. VV-ECMO can stabilize patients with life-threatening respiratory failure and bridge them to definitive diagnostic evaluation. NUT carcinoma should be considered in young patients with rapidly progressive thoracic masses and poorly differentiated malignancy, with early confirmatory immunohistochemical or molecular testing.

## Introduction

Nuclear protein in testis (NUT) carcinoma is a rare and highly aggressive malignancy defined by rearrangement of the *NUTM1 *gene on chromosome 15q14. The most common genetic alteration is fusion of *NUTM1 *with *BRD4 *on chromosome 19p13.12, although other fusion partners have been described. The resulting oncoprotein promotes dysregulated transcription and blocks cellular differentiation, leading to rapid tumor progression and poor clinical outcomes [1,2]. Historically referred to as NUT midline carcinoma, this entity can arise in various anatomic locations but most commonly involves midline structures of the thorax, head, and neck [3].

NUT carcinoma is associated with particularly poor survival and frequently presents with advanced or metastatic disease [4,5]. Clinical manifestations are nonspecific and may include cough, dyspnea, chest pain, hemoptysis, or symptoms related to compression of mediastinal structures [4]. Because of its rarity and poorly differentiated histologic appearance, NUT carcinoma is often initially misclassified as poorly differentiated nonsmall cell lung carcinoma, squamous cell carcinoma, or other undifferentiated malignancies [6].

Obtaining a diagnosis may be especially challenging when patients present with large mediastinal masses causing significant airway compromise. We report a case of NUT carcinoma in a young woman who developed catastrophic hypoxemia during anesthetic induction for diagnostic bronchoscopy and therapeutic tumor debulking, requiring emergent venovenous extracorporeal membrane oxygenation (VV-ECMO) as rescue therapy to stabilize respiratory failure and permit completion of the diagnostic evaluation.

## Case presentation

A 29-year-old woman with morbid obesity (body mass index: 51.2 kg/m²) and polycystic ovarian syndrome treated with metformin presented with several weeks of progressive fatigue, dyspnea, and hemoptysis. She initially tested positive for COVID-19 in the outpatient setting and was managed conservatively; however, persistent and worsening respiratory symptoms prompted treatment with antibiotics and corticosteroids for presumed superimposed pneumonia. She was subsequently admitted to an outside hospital for progressive hypoxemia and further evaluation. Initial diagnostic workup, including thoracentesis, was nondiagnostic, and she was transferred to our institution for additional evaluation and tissue diagnosis.

**Figure 1:**
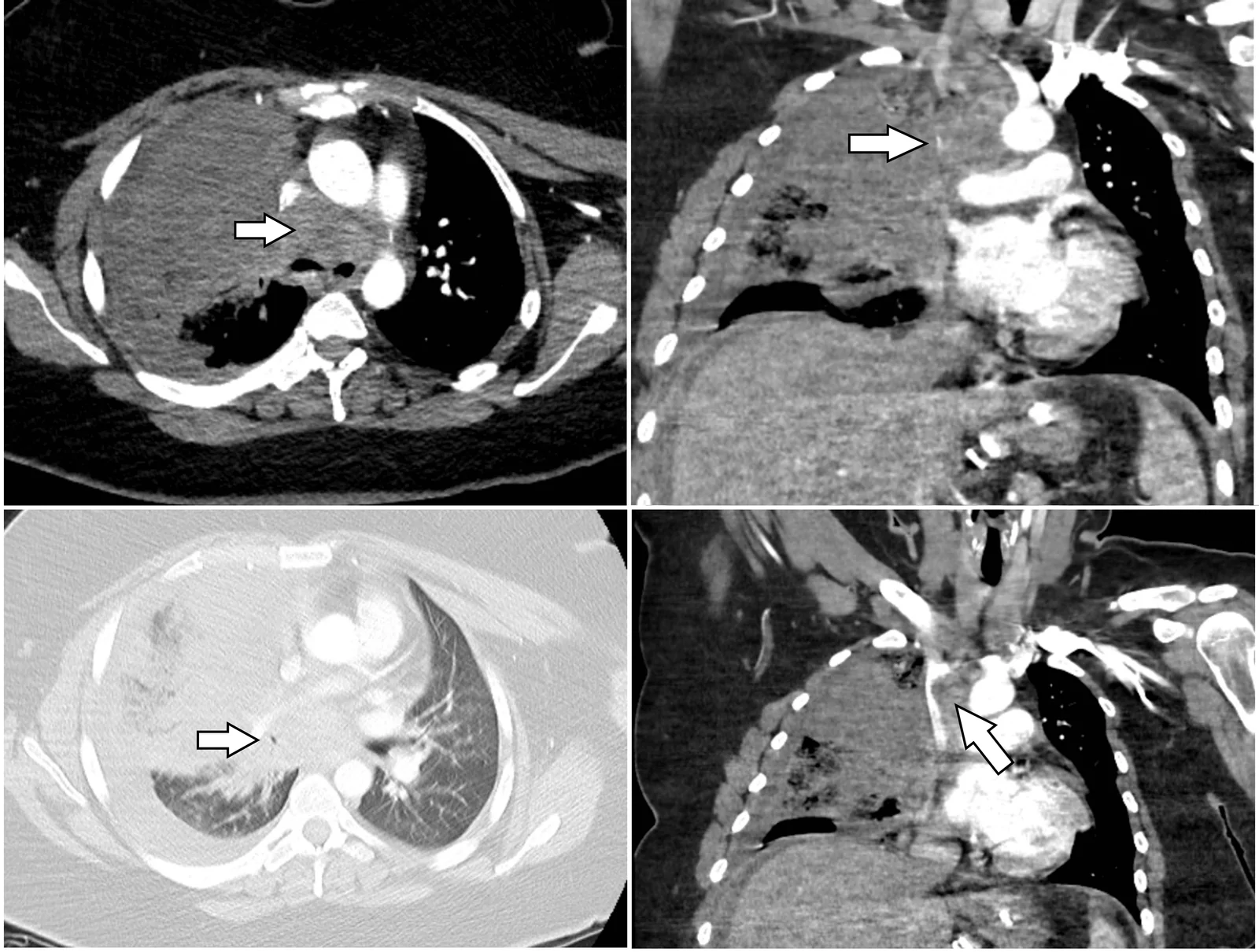
Contrast-enhanced computed tomography of the chest demonstrates a large right superior mediastinal mass. The top left (axial) and top right (coronal) images show the bulky mediastinal mass (white arrows) causing marked compression and near-complete effacement of the superior vena cava (SVC). The bottom left (axial) image demonstrates severe compression of the right mainstem bronchus (white arrow). The bottom right (coronal) image demonstrates near-complete SVC obstruction secondary to extrinsic compression by the mediastinal mass (white arrow).

On initial examination, the patient appeared visibly dyspneic with near-absent breath sounds over the right hemithorax. She was hypoxemic and required escalating supplemental oxygen via a nasal cannula, up to 10 L/minute, to maintain adequate oxygenation. A pulmonary embolism-protocol computed tomography (CT) scan demonstrated a large right hilar and mediastinal mass compressing the trachea, pulmonary artery, and superior vena cava (SVC) (Figure 1). Given the patient’s young age and imaging findings, the differential diagnosis included lymphoma, thymic malignancy, germ cell tumor, poorly differentiated primary lung carcinoma, and metastatic carcinoma. Establishing a tissue diagnosis was essential to guide treatment.

**Figure 2:**
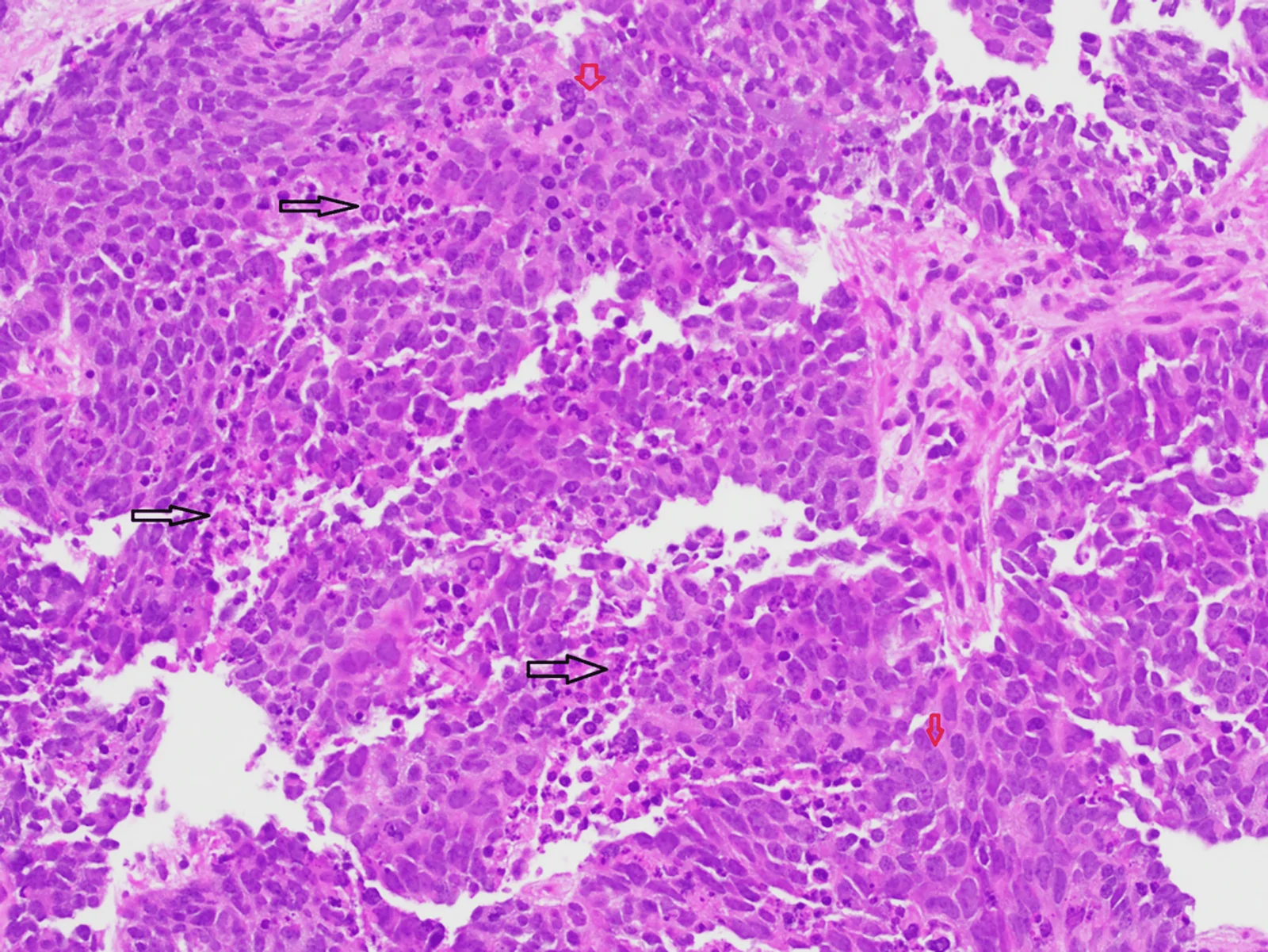
Hematoxylin and eosin stain (40×) showing sheets of poorly differentiated cells with a high nuclear-to-cytoplasmic ratio. The cells show focal prominent nucleoli (red arrows) and increased karyorrhexis (black arrow), suggestive of highly proliferative, poorly differentiated malignancy.

**Figure 3:**
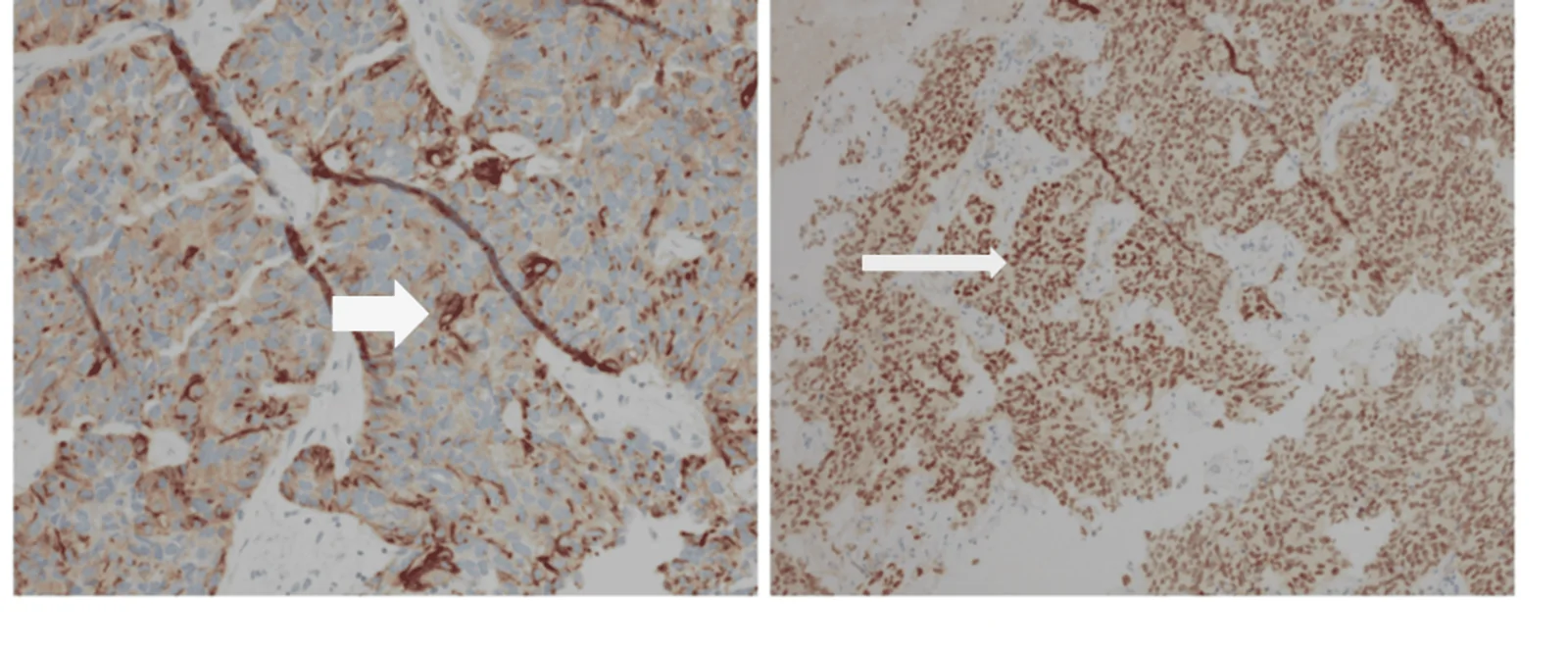
Immunochemistry staining of supraclavicular lymph node. Left: Cells (200×) staining patchy positive for AE1/AE3 and CK7 (white thick arrow), along with focal weak expression of synatophysin. Right: Cells (100×) also staining positive for NUT (white thin arrow) confirming the diagnosis of NUT carcinoma.

The decision was made to pursue rigid bronchoscopy because of her ongoing hemoptysis, significant central airway compression, and the potential need for therapeutic intervention should a removable endobronchial obstruction be identified, in addition to obtaining diagnostic tissue. During induction of anesthesia for rigid bronchoscopy, she developed profound and persistent hypoxemia despite airway optimization and placement of the rigid bronchoscope. The clinical deterioration was attributed to worsening dynamic airway compression from the mediastinal mass, resulting in severe impairment of ventilation that could not be corrected with conventional airway maneuvers. Despite attempts at ventilation optimization, including jet ventilation, adequate oxygenation could not be achieved, prompting emergent initiation of VV-ECMO for rescue of gas exchange and stabilization. Bronchoscopy revealed 40-50% stenosis of the right mainstem bronchus due to extrinsic compression and a large friable endobronchial mass causing complete occlusion of the right upper lobe bronchus. Because the patient’s critical airway compromise was predominantly due to extrinsic compression of the central airway rather than a removable endobronchial lesion, no therapeutic bronchoscopic intervention was performed. Following stabilization with VV-ECMO, endobronchial ultrasound-guided transbronchial needle aspiration and biopsies of the hilar mass and mediastinal lymph nodes were performed but were nondiagnostic because of extensive necrosis and crush artifact. Subsequent positron emission tomography-computed tomography (PET-CT) demonstrated widespread hypermetabolic disease, including supraclavicular lymph node involvement. Percutaneous biopsy of a supraclavicular lymph node demonstrated metastatic poorly differentiated carcinoma composed of poorly differentiated epithelioid cells with high nuclear-to-cytoplasmic ratios, vesicular nuclei, prominent nucleoli, and extensive necrosis. Tumor cells showed diffuse nuclear NUT immunoreactivity with patchy AE1/AE3 and CK7 positivity and focal synaptophysin expression, confirming NUT carcinoma (Figures 2, 3).

Following completion of bronchoscopy and additional tissue acquisition, the patient’s oxygenation gradually improved with supportive care, allowing successful extubation followed by VV-ECMO decannulation several days later. Importantly, ECMO decannulation reflected improvement of the acute procedural respiratory failure rather than resolution of the underlying tumor-related airway compromise. No definitive airway intervention or tumor-directed therapy had been performed before decannulation. Unfortunately, her condition rapidly declined before she could initiate cisplatin-based chemotherapy. She developed refractory hypoxemic respiratory failure requiring re-intubation and high-level mechanical ventilatory support, septic shock requiring multiple vasopressors, tumor lysis syndrome, renal failure, and eventually cardiac arrest. She passed away on hospital day 29 after transitioning to comfort care measures only. Table 1 presents a summary of the patient presentation.

**Table 1:** Timeline illustrating the patient’s clinical progression from initial presentation with respiratory symptoms to identification of a large mediastinal mass, development of peri-procedural respiratory failure requiring venovenous extracorporeal membrane oxygenation, subsequent diagnostic evaluation, confirmation of NUT carcinoma by supraclavicular lymph node biopsy, and eventual clinical deterioration despite supportive management. COVID-19 = coronavirus disease 2019; CT = computed tomography; ICU = intensive care unit; IHC = immunohistochemistry; PEA = pulseless electrical activity; PET = positron emission tomography; VV-ECMO = venovenous extracorporeal membrane oxygenation; NUT = nuclear protein in testis

Hospital day	Clinical phase and setting	Clinical events and diagnostic findings
Day −53	Initial presentation - outpatient clinic	Outpatient presentation with cough and dyspnea; confirmed COVID-19 positive
Day −27	Persistent symptoms - outpatient clinic	Returned to clinic with persistent respiratory symptoms. Treated empirically with antibiotics and corticosteroids for presumed pneumonia
Day 0	Acute emergency department presentation - emergency department	Presented to the emergency department with progressive dyspnea, new-onset hemoptysis, and worsening hypoxemia. CT of the chest revealed a large mediastinal mass with significant airway and vascular compression
Day 2	Diagnostic workup - inpatient ward	Diagnostic thoracentesis was performed; cytologic evaluation did not identify evidence of primary malignancy
Day 4	Tertiary transfer - tertiary care center	Transferred to a tertiary care center for further evaluation, including advanced bronchoscopic assessment and tissue diagnosis
Day 7	Airway emergency and ECMO - operating room	Underwent rigid bronchoscopy for biopsy. Developed profound refractory hypoxemia prompting emergent VV-ECMO cannulation for respiratory rescue. Underwent bronchoscopy for initial tissue biopsy that was non-diagnostic
Day 9	ICU recovery - intensive care unit	Successfully extubated while receiving VV-ECMO support
Day 10	ECMO decannulation - intensive care unit	VV-ECMO was successfully discontinued following improvement in respiratory status
Day 11	Staging assessment radiology - intensive care unit	PET-CT demonstrated widespread hypermetabolic disease, including hypermetabolic disease in a supraclavicular lymph node
Day 14	Tissue acquisition interventional radiology	Interventional radiology performed a supraclavicular lymph node biopsy for definitive tissue diagnosis
Day 18	Definitive diagnosis pathology	Pathology confirmed NUT carcinoma based on positive nuclear NUT immunohistochemical staining
Day 20	Clinical deterioration - intensive care unit	Developed clinical deterioration with tumor lysis syndrome and renal failure in the setting of progressive malignancy
Day 22	Cardiac arrest - intensive care unit	Experienced PEA cardiac arrest, suspected to be multifactorial, including progressive hypoxemia and disease burden
Day 29	Goals of care palliative care	Due to continued clinical deterioration, the family elected to transition to comfort-focused care, and the patient subsequently died

## Discussion

NUT carcinoma remains one of the most aggressive thoracic malignancies and presents diagnostic challenges due to its nonspecific clinical presentation, poorly differentiated histologic features, and frequent need for specialized molecular or immunohistochemical testing. Patients frequently present with nonspecific respiratory symptoms such as cough, dyspnea, chest pain, and hemoptysis, often leading to delays in diagnosis [4]. Radiographic findings such as pulmonary infiltrates, consolidative changes, pleural effusions, or a centrally located thoracic mass may initially be attributed to infectious or inflammatory processes, particularly in young patients without traditional risk factors for lung cancer. Consequently, diagnosis is often delayed [6].

The diagnosis of NUT carcinoma requires a high index of suspicion. Histologically, tumors are typically poorly differentiated and may resemble other thoracic malignancies, including poorly differentiated squamous cell carcinoma, undifferentiated nonsmall cell lung carcinoma, small cell carcinoma, or other round cell neoplasms [1,4]. Nuclear staining for NUT protein by immunohistochemistry serves as an effective screening tool, while molecular confirmation of *NUTM1* rearrangement may be performed using fluorescence in situ hybridization or next-generation sequencing when available [7].

This case also highlights the challenges associated with establishing a diagnosis in rapidly growing thoracic malignancies. Although NUT immunohistochemistry provided a definitive diagnosis once performed, initial diagnostic evaluation was limited by extensive tumor necrosis and crush artifact, resulting in nondiagnostic bronchoscopic and endobronchial ultrasound-guided biopsies. Initial bronchoscopic and endobronchial ultrasound-guided biopsies were nondiagnostic because of extensive necrosis and crush artifact. Tumor necrosis has been identified as an important contributor to reduced diagnostic yield in mediastinal and thoracic biopsies [8,9]. In patients with highly aggressive tumors, repeat sampling from metabolically active regions identified on PET may improve diagnostic success [10].

An additional teaching point is the management of patients with large mediastinal masses undergoing diagnostic procedures. Compression of central airways and major vascular structures increases the risk of catastrophic respiratory or hemodynamic collapse during sedation or general anesthesia [11]. Careful preprocedural planning and multidisciplinary coordination are therefore essential.

VV-ECMO provided a critical bridge in this case, permitting stabilization after severe hypoxemia developed during anesthetic induction and allowing completion of bronchoscopic airway evaluation and tissue sampling. VV-ECMO served as temporary respiratory support until further diagnostic evaluation could be completed, ultimately allowing definitive diagnosis through supraclavicular lymph node biopsy. Although ECMO is most commonly used for refractory respiratory failure, growing evidence supports its role as rescue therapy in selected patients who develop life-threatening airway compromise during diagnostic or therapeutic procedures [12,13]. In select patients who develop life-threatening airway compromise during diagnostic or therapeutic procedures, extracorporeal support may provide a temporary rescue strategy to facilitate stabilization.

The prognosis of NUT carcinoma remains poor despite advances in molecular characterization. Most patients present with locally advanced or metastatic disease, and reported survival remains measured in months [4,5]. Platinum-based chemotherapy, radiation therapy, and immune checkpoint inhibitors have demonstrated limited benefit. Novel targeted approaches, including bromodomain and extraterminal domain inhibitors, remain under active investigation [1,14].

## Conclusions

NUT carcinoma is a rare and highly aggressive malignancy that should be considered in young patients presenting with a rapidly progressive mediastinal or hilar mass or poorly differentiated carcinoma on initial evaluation. Diagnosis is frequently delayed due to nonspecific clinical presentation. Advanced disease at presentation and extensive tumor necrosis may limit the diagnostic yield of initial biopsies. Early use of NUT immunohistochemistry, followed by confirmatory molecular testing when available, is essential for timely diagnosis. Patients with large mediastinal masses are at high risk for airway obstruction and hemodynamic collapse during sedation or general anesthesia. This case highlights the importance of multidisciplinary planning for high-risk airway procedures and demonstrates that VV-ECMO may serve as rescue support in select patients who develop life-threatening respiratory compromise during airway instrumentation, allowing stabilization and completion of the diagnostic evaluation. Despite advances in molecular characterization, outcomes for thoracic NUT carcinoma remain poor, underscoring the need for earlier recognition, improved diagnostic strategies, and continued development of effective targeted therapies.
